# Exploiting full-duplex opportunities in WLANs via a reinforcement learning-based medium access control protocol

**DOI:** 10.1038/s41598-024-83025-y

**Published:** 2024-12-28

**Authors:** Song Liu, Peng Wei

**Affiliations:** 1 Naval University of Engineering, Wuhan, Hubei China; 2 National University of Defense Technology, Changsha, Hunan China; 3 National Key Laboratory on Ship Vibration & Noise, Wuhan, China

**Keywords:** In-band full duplex, Medium access control protocol, Reinforcement learning, Bayesian optimization, Multi-armed bandit, Electrical and electronic engineering, Computer science

## Abstract

In-band full-duplex communication has the potential to double the wireless channel capacity. However, how to efficiently transform the full-duplex gain at the physical layer into network throughput improvement is still a challenge, especially in dynamic communication environments. This paper presents a reinforcement learning-based full-duplex (RLFD) medium access control (MAC) protocol for wireless local-area networks (WLANs) with full-duplex access points. To solve the channel contention problem and fully utilize the full-duplex transmission opportunities, we first design a two-way handshake transmission mechanism and make an investigation on the effects of transmission scheduling in full-duplex transmission. Then the transmission scheduling problem is theoretically formulated as a non-stationary multi-armed bandit problem in which our objective is to maximize the network throughput. Thus, we develop a Window-Constraint Bayesian (WCB) algorithm to generate optimized scheduling policies online. And full-duplex opportunities are fully utilized by transmitting packets according to the optimized scheduling policies. Besides, an analytical model is developed to characterize the performance of RLFD. The performance of RLFD is evaluated by simulation. And the results show that RLFD can improve the network throughput by 80% compared with the IEEE 802.11 distributed coordination function with Request-To-Send/Clear-To-Send. Moreover, with the proposed WCB algorithm, the network throughput can remain steady as the communication environment dynamically changes.

## Introduction

With the explosive growth of data traffic in edge-area wireless networks, current wireless communication systems are facing great challenges^1^. Especially for a wireless local-area network (WLAN) with an access point (AP), all data traffic in the network needs be exchanged at the AP. If the AP is saturated, the network will be blocked. To improve the network performance while maintaining compatibility with legacy wireless end devices, this paper applies the in-band full-duplex (FD) technique to APs and keeps clients working in half-duplex (HD) mode. With in-band full-duplex techniques, wireless devices can receive and send packets simultaneously in the same frequency band, which is impossible with legacy half-duplex (HD) wireless devices because of strong self-interference^2^. Fortunately, recent works have demonstrated that the full-duplex radio is practical^3,4^. Thus, full-duplex APs are anticipated to double the network capacity and significantly improve the network performance in a WLAN. However, how to make use of full-duplex communication opportunities to convert the full-duplex advantage in the physical (PHY) layer into the network performance improvement in the medium access control (MAC) layer is still a challenge.

**Fig. 1:**
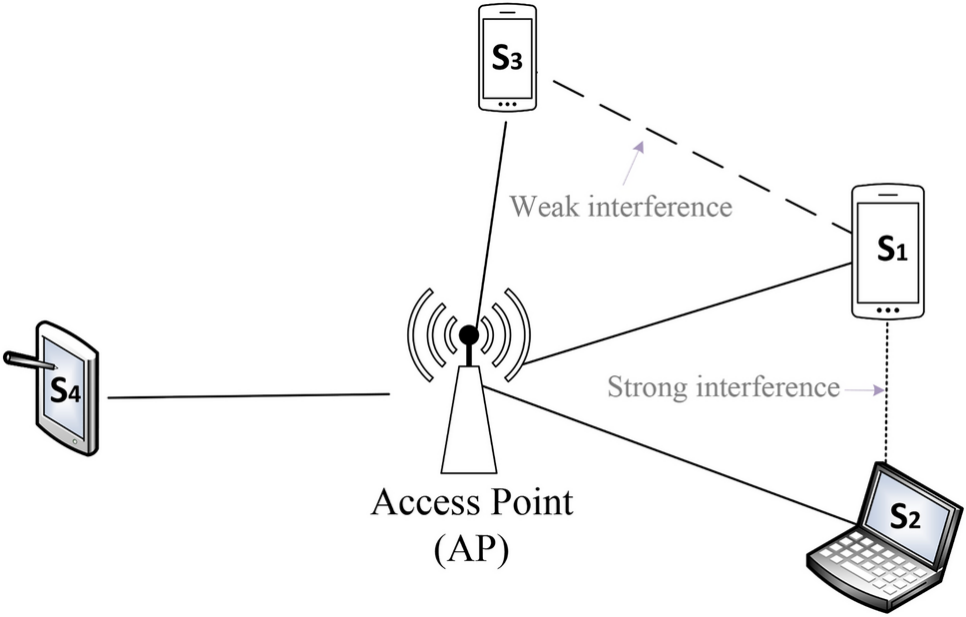
An illustrative example for exploiting full-duplex opportunities.

The first issue is how to exploit potential asymmetric full-duplex transmission opportunities in dynamic environments. Asymmetric transmissions are transmissions among three nodes, such as $S_1 \rightarrow AP \rightarrow S_4$ as shown in Fig. 1. While $S_1$ uploads packets to AP, AP can download packets to other clients. But how the AP selects the downlink client in the asymmetric transmission determines whether the full-duplex opportunity can be effectively utilized. For example, if AP selects $S_2$ as the downlink client, the uplink transmitting signal from client $S_1$ will interfere with the downlink signal at $S_2$ from AP. If AP selects $S_4$ as the downlink client, asymmetric full-duplex transmission $S_1 \rightarrow AP \rightarrow S_4$ can be utilized since the uplink signal from $S_1$ will not interfere with the downlink signal at $S_4.$ To address the downlink client selection problem in asymmetric transmissions, there are mainly three methods: (1) AP randomly selects a downlink client^5–7^, which can only obtain low full-duplex opportunities and the network performance improvement is limited. (2) AP selects a downlink client based on historical information^8,9^. For example, in the last *n* transmissions, while $S_1$ uploads packets, AP chooses $S_2$ and $S_3$ as the downlink client can obtain a performance improvement of 30% and 70%, respectively. Then $S_1$ will choose $S_3$ as the downlink client. However, this greedy method may miss better choices since the best choices may not yet present in historical data. (3) AP selects a downlink client based on network measurements^10,11^, in which AP firstly sends a probe packet and all clients reply to the packet so that the interference information can be gathered. Then an optimized transmission scheduling policy can be generated. This approach introduces additional measurement overhead. In addition, node mobility and uncertain interference can lead to dynamic changes in wireless channels, resulting in outdated interference information based on history and measurements. Therefore, how to effectively schedule asymmetric transmissions in the dynamic communication environment is still a challenging problem.

The second issue is how to efficiently solve the hidden/exposed terminal problems. The hidden and exposed terminal problems are fundamental in traditional wireless communication and aggravated in full-duplex communication^12^. For example, as shown in Fig. 1, while AP sends packets to $S_4,$ $S_1$ can upload packets to the AP since AP works in full-duplex mode. But $S_1$ cannot do that because it senses and finds that the wireless channel is busy, which can be seen as a new case of exposed terminal problem in full-duplex communication. In another example, if an AP is sending packets in the full-duplex transmission $S_1 \rightarrow AP \rightarrow S_4,$ $S_4$’s neighbor (connected with another AP) also sends packets at the same time since it cannot overhear the signal from $S_4$’ AP and believes that the wireless channel is available. Unfortunately, $S_4$ can receive its neighbor’s signal, resulting in the failure of the full-duplex transmission. The hidden and exposed problems are usually solved by the Request-To-Send/Clear-To-Send (RTS/CTS) mechanism. However, most existing full-duplex MAC protocols based on the Carrier Sense Multiple Access (CSMA) mechanism assume that both APs and clients are equipped with full-duplex radios^9,12^. In addition, few full-duplex MAC protocols pay attention to the exposed terminal problem when an AP preempts the wireless channel, and how to initiate full-duplex transmission in this case. Therefore, it is necessary to further optimize the full duplex transmission mechanism to improve the opportunities for full duplex communication while addressing hidden terminals.

The third issue is how to discover and utilize full-duplex opportunities in a distributed manner. So far, many full-duplex MAC protocols have been proposed, among which centralized protocols can greatly improve the network performance, such as pFD-MAC^10^, Janus^11^. In these centralized full-duplex MAC protocols, an optimized full-duplex transmission scheme can be efficiently generated according to the traffic and interference information, so that they can obtain good network performance. But centralized scheduling will also introduce scalability and reliability issues. The CSMA mechanism is the defacto standard and has been widely used in half-duplex wireless communication. However, owing to the limitation of the CSMA mechanism, these protocols can hardly resolve the channel contention problem in full-duplex wireless networks^8,13^, resulting in a low channel utilization rate. For example, A-Duplex^8^ is a typical CSMA-based full-duplex MAC protocol and supports half-duplex clients, but its channel utilization rate can only reach 86% while the upper limit could be 200%. Therefore, how to design a distributed full-duplex MAC protocol based on the CSMA mechanism to maximize the network performance still needs further research.

Recently, reinforcement learning (RL) has been successfully used solve decision-making and optimization problems in wireless networks^14–18^. The key ideal behind RL is that an agent can learn to make decisions by receiving feedback in the form of rewards, and balance between exploiting what is known to maximize immediate performance and exploring what is unknown to improve future performance. For example, Liu et al.^14^ use RL to balance energy and routing in wireless sensor networks, which can successfully extend the lifetime of the network. Ren et al.^16^ characterize the full-duplex transmission procedure of ad-hoc networks into Markov decision processes (MDP), and proposed a Q-learning algorithm to reduce inter-node interference in the network and optimize the network throughput. Yu et al.^19^ proposed a deep RL-based protocol to learn an optimal channel access strategy to achieve a certain pre-specified global objective for heterogeneous wireless networks. Motivated by these works, we can also leverage the advantages of RL to improve scheduling efficiency in full duplex transmission, and enable it to actively adapt to dynamic environments.

Therefore, to address the above issues and overcome the shortcomings of previous approaches, this paper studies the full-duplex transmission scheduling problem in a dynamic wireless communication environment and presents a reinforcement learning-based MAC protocol for AP-based full-duplex WLANs, called RLFD. The RLFD MAC protocol is designed based on the CSMA mechanism and operates in a distributed manner. To make full use of full-duplex transmissions, a novel two-way handshake transmission mechanism is first proposed, and it can efficiently solve the hidden/exposed terminal problems. Then, the full-duplex transmission scheduling problem in a dynamic environment is formulated as a multi-armed bandit (MAB) problem, in which its objective is to maximize the network throughput. Thus, we develop a Window-Constrained Bayesian (WCB) algorithm to schedule transmissions online, and theoretically demonstrate that the algorithm can achieve optimized scheduling while ensuring fast convergence in dynamic environments. By transmitting according to the optimized scheduling policy, potential full-duplex opportunities can be efficiently exploited. Extensive simulation results have verified the effectiveness of the proposed protocol. And compared with traditional IEEE 802.11 distributed coordination function (DCF) with RTS/CTS, our proposed RLFD protocol can improve the network throughput by 80%. Moreover, with the proposed WCB algorithm, the network throughput remains steady as the communication environment dynamically changes.

The main contributions of this paper can be summarized as:

- We study the full-duplex transmission scheduling problem in the dynamic communication environment and propose a reinforcement learning-based full-duplex MAC protocol named RLFD. The RLFD MAC protocol has backward compatibility and supports the coexistence of half-duplex clients and full-duplex APs.
- We make an investigation on how full-duplex scheduling affects network performance and design a novel two-way handshake transmission method based on the CSMA mechanism to solve the channel contention problem. An analytical model based on the Markov-chain is also developed to analyze the performance of the proposed protocol.
- The full-duplex transmission scheduling problem in a dynamic communication environment is formulated as a non-stationary MAB problem. To solve the problem, we develop a Window-Constrained Bayesian algorithm and theoretically demonstrate that the algorithm can obtain an optimized scheduling policy while ensuring fast convergence in dynamic communication environments.
- Extensive simulations are conducted to validate the effectiveness of the proposed protocol. And the results indicate that RLFD can significantly improve the network throughput and remain steady in dynamic communication environments.

## Related work

To date, there have been many studies on wireless full-duplex MAC protocols. Singh et al.^9^ designed the first distributed full-duplex protocol ContraFlow based on the Carrier Sense Multiple Access/Collision Avoidance (CSMA/CA) mechanism. And historical information was used to allow the receiving node to initiate a second transmission in ContraFlow. Goyal et al.^20^ designed a distributed full-duplex MAC protocol based on the CSMA/CA mechanism and used a handshake mechanism to determine whether full-duplex transmission can be initiated by adding two flag bits. Liao et al.^21^ proposed a full-duplex protocol FD-MAC, which is similar to the Carrier Sense Multiple Access/Collision Detection (CSMA/CD) mechanism. FD-MAC mainly avoids performance losses caused by frame conflicts by using the full-duplex radio to monitor interference in wireless channels. But it does not utilize any full-duplex transmission. These protocols only utilize full-duplex radio to improve network performance, without considering the issue of hidden terminal, resulting in limited performance improvement.

A-Duplex^8^ utilizes capture effects to establish full-duplex transmissions based on IEEE 802.11 DCF with RTS/CTS. But it only supports full-duplex transmission initiated by the uplink client, and cannot utilize full-duplex opportunities when the AP first preempts the wireless channel. AUB protocol^22^ uses an idle uplink period to transmit an acknowledgement frame and report the buffer information. Then, the AP can attempt to schedule the buffer information of the nodes accordingly without competition. ASF-MAC^23^ considers both downlink-based and uplink-based initiated full-duplex transmission issues. But for downlink-based initiated full-duplex transmission, when the AP first preempts the channel, clients need to compete for the uplink transmission using CSMA/CA, which is inefficient and prone to failure in initiating the full-duplex transmission. FDOE^24^ leverages rateless coding and pseudo-noise sequences to establish a full-duplex transmission in wireless mesh networks. These protocols mainly focus on utilizing full-duplex transmission by optimizing the design of transmission mechanisms and few work studies on the link scheduling methods.

In fact, there are also many studies on traffic scheduling and link scheduling in wireless networks. But many of them focus on end-to-end transmission optimization^25–27^. For example, Tu et al.^28^ designed an efficient multi-flow multicast transmission algorithm to increase the number of concurrent multimedia flows, Wang et al.^15^ applied the double Q-learning algorithm to optimize end-to-end transmission latency. However, the link scheduling in full-duplex MAC protocols mainly focuses on the inter-node inference issue. Tong et al.^29^ utilized the theory of multiplayer MAB theory to solve the self-interference problem in full-duplex networks by intelligently adjusting parameters such as transmit power and carrier perception threshold, thereby improving the network performance. Wilhelmi et al.^30^ used the MAB theory in the transmit power selection and channel selection issue in WLAN to optimize the network performance.

Inspired by these works, we will apply reinforcement learning theory to solve the full-duplex transmission scheduling problem in the AP-based wireless network. We will propose an efficient scheduling algorithm to fully utilize the full-duplex opportunities and design an efficient transmission mechanism to carry out the scheduling policy.

## System model and problem description

### System model

#### Network and physical model

This paper considers an AP-based or infrastructure-based wireless network model, in which APs are equipped with full-duplex radios and clients operate in half-duplex mode. Since only AP works in full-duplex mode, asymmetric transmissions can be utilized only via the AP in a network, that is, transmissions between three nodes, such as the transmission $S_1 \rightarrow AP \rightarrow S_4$ as shown in Fig. 1. And no symmetric full-duplex transmissions, that is, transmissions between two nodes, such as $S_1 \leftrightarrow AP,$ can occur. For the convenience of description, we refer to the transmission from clients to AP as uplink transmission and the transmission from AP to clients as the downlink transmission. In addition, considering that the full-duplex radio can achieve a capacity gain of 1.95*X* over half-duplex mode in physical layer^31^, we assume perfect self-interference cancelation (SIC) technique can be applied and mainly focus on the network-level capacity.

#### Propagation model

We assume the transmitting signal between the transmitter and receiver is Rayleigh faded, which is widely adopted to characterize the propagation of the indoor wireless signal. Then, the power of the received signal can be calculated as:

1 $$\begin{aligned} P_r = \kappa P_t h r^{-n}, \end{aligned}$$

where $P_r$ is the received signal strength and $P_t$ is the transmitted signal strength. $\kappa$ stands for the propagation constant, *h* is the channel coefficient and *r* is the distance between the receiver and transmitter. *n* is the pass loss exponent.

In an asymmetric transmission, downlink transmission may interfere with the uplink transmission. To express the inter-client interference relationship among clients, signal-to-interference plus noise ratio (SINR) is used. Assume that there is an asymmetric transmission between client $S_i$ and client $S_j$ ($S_i \rightarrow AP \rightarrow S_j$), we use $c_{ij}$ to express the SINR value at client $S_j$ and it can be calculated by:

2 $$\begin{aligned} c_{ij} = P_r(0,i)/(P_r(i,j) + P_N), \end{aligned}$$

in which $P_r(0,i)$ represents the received signal strength at client $S_i$ from AP and $P_r(i,j)$ represents interference signal strength at $S_j$ from client $S_i.$ $P_N$ indicates the noise signal strength. The value of $c_{ij}$ can also be measured in the wireless network using the received signal strength indicator (RSSI).

#### Multi-rate transmission support

Wireless devices can choose different modulation methods to transmit signals under different SINR conditions, and different modulation methods correspond to different data transmission rates. In this paper, we assume that wireless devices support multi-rate transmission. And the transmission data rate can be adjusted according to the wireless communication environment. The higher the SIR value, the better the communication environment and higher data rate can be chosen.

### Problem description

To maximize the network throughput, two aspects need to be considered: (1) design an efficient full-duplex transmission scheduling algorithm to fully explore and utilize the full-duplex opportunity; (2) provide an effective packet transmission mechanism to carry out the scheduling algorithm. Here, we first discuss the full-duplex transmission scheduling problem. Considering that only APs work in full-duplex mode, asymmetric transmissions are the only full-duplex opportunity that can be utilized. Thus, in the full-duplex transmission scheduling problem, maximizing network throughput is equivalent to maximizing the network performance gain brought by asymmetric transmissions.

In asymmetric transmission, the network performance gain is mainly determined by the downlink transmission. Due to APs operating in full-duplex mode, the uplink signal can achieve perfect SIC at the AP. Thus, the performance of uplink transmission will not be affected regardless of whether there is a downlink transmission. However, the uplink will interfere with the downlink, and the interference from the same uplink on different downlinks will be different, so different asymmetric transmissions will also have different performances. Assume that there are *N* clients and one AP in the network, when a client $S_i$ $(i = 1,2,...,N)$ uploads packets to AP, the AP has $N-1$ options to select the downlink transmission. Without loss of generality, assume that AP selects $S_j$ ($j = 1,2,...,N$ and $j \ne i$) as the downlink client. Then, after asymmetric transmission $S_i \rightarrow AP \rightarrow S_j$ is over, full-duplex transmission gain is obtained, denoted by $R^i_j(t),$ in which *t* means the time step. When the system runs for some time, the cumulative network performance gain obtained by client $S_i$ can be expressed as $G_i = \sum _t \sum _j R^i_j (t).$ Then the overall network performance gain can be calculated by:

3 $$\begin{aligned} G_t = \sum ^N_{i=1} G_i = \sum ^N_{i=1} \sum _t \sum ^N_{j=1,j \ne i} R^i_j (t) = \sum _t \sum ^N_{i=1} \sum ^N_{j=1,j \ne i} R^i_j (t). \end{aligned}$$

Then, the full-duplex transmission scheduling problem can be expressed as maximizing the network performance gain over some time by optimizing the downlink selection policy:

4 $$\begin{aligned} \max G_t = \max \sum _t \sum ^N_{i=1} \sum ^N_{j=1,j \ne i} R^i_j (t). \end{aligned}$$

If the interference information of the network is known, we can calculate the performance gain $R^i_j$ for each asymmetric transmission. When client $S_i$ needs to upload data to AP, AP only needs to select $S_j$ with maximum performance gain $\max \{R^i_j\}$ to initiate asymmetric transmission. However, considering the dynamic changes in the communication environment due to client mobility and unknown interference in wireless networks, it is difficult for nodes to obtain all interference information in advance. In fact, we can understand this problem from the perspective of reinforcement learning. The performance gain can be regarded as a reward signal, and nodes need to get to know the environment by interacting with it and then make the optimal choice. For each client node, there are N-1 choices. It needs to exploit the best of what it has experienced, but it also needs to explore to make better choices. Therefore, selecting the downlink client in asymmetric transmissions can be seen as a $(N-1)$-armed bandit problem. Moreover, considering the dynamic nature of the wireless communication environment, the *Reward* is not a stationary value for asymmetric transmission. Thus, the transmission scheduling problem can be seen as a non-stationary multi-armed bandit problem.

## The framework of RLFD protocol

**Fig. 2:**
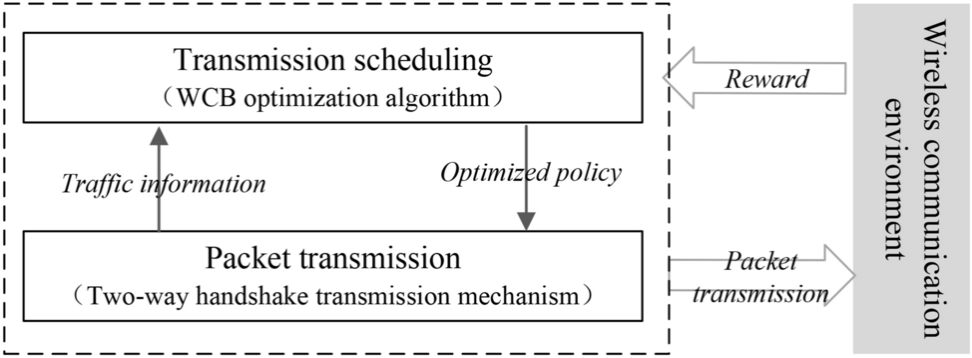
The workflow of the proposed RLFD protocol.

The proposed RLFD protocol mainly consists of two parts: transmission scheduling and packet transmission, as shown in Fig. 2. The transmission scheduling part is responsible for discovering and utilizing full-duplex opportunities, while the packet transmission part is responsible for reliable transmission and solving problems such as hidden terminals and exposed terminals. The packet transmission part will tell the transmission scheduling part the traffic information, such as who has preempted the wireless channel and attempted to start a full-duplex transmission. The transmission scheduling part will return the optimized policy, that is, the optimized full-duplex transmission can be utilized. When the full-duplex transmission is over, AP can obtain a *Reward* and the parameters in WCB algorithm can be updated accordingly. The details of transmission scheduling and packet transmission will be introduced in “The WCB algorithm for transmission scheduling” and “Two-way handshake transmission mechanism”, separately. Besides, we have also developed an analytical model to analyze the performance of RLFD protocol with saturated traffic, which is described in Section “Performance analysis”.

## The WCB algorithm for transmission scheduling

As mentioned in above section, the full-duplex transmission scheduling problem can be seen as a non-stationary MAB problem, and it also has an exploration-exploitation dilemma. On the one hand, facing a dynamic unknown wireless communication environment, exploration is needed to learn the performance gain of each full-duplex transmission. On the other hand, exploitation of the optimal full-duplex transmission to maximize network throughput is also necessary. Accordingly, we propose a WCB algorithm based on Thompson Sampling to solve the asymmetric transmission scheduling problem.

### Reward

In reinforcement learning, the optimization goal is to maximize the cumulative *reward* value over a period of time. Therefore, the design of *reward* signal is the key to achieving optimization goals. To maximize the network throughput, we use the theoretical channel capacity as the *reward* signal in our proposed algorithm. And there are three reasons: (1) Generality. Different wireless communication technologies with different encoding or modulation methods may result in varying network performance. However, a specific wireless channel capacity has a limit, which is given by Shannon’s Theorem. And all existing communication technologies are constantly narrowing the gap with this limit. (2) Accuracy. The network throughput is determined by the data rate and packet loss rate, and they are both determined by the SINR value in a certain communication environment. Thus, the theoretical channel capacity can accurately express our optimization goal—network throughput. (3) Easy to measure. In the MAC protocol, the RSSI value can be easily obtained during the RTS/CTS handshake process, and then the SINR value can be calculated. Therefore, for an asymmetric transmission $S_i \rightarrow AP \rightarrow S_j,$ we use the theoretical channel capacity of the downlink transmission as the *reward* signal $R^i_j$:

5 $$\begin{aligned} R^i_j = log_2(1+c_{ij})/log_2(1+c_{max}), \end{aligned}$$

where $c_{ij}$ is the SINR value at downlink client $S_j$ in the asymmetric transmission $S_i \rightarrow AP \rightarrow S_j,$ and $c_{max}$ is the maximum SINR value in the wireless communication.

When using the Bayesian optimization framework with Beta functions, it is also necessary to consider the design of the *Regret* function. Considering that the downlink selection in an asymmetric transmission may not be the optimal choice, the *Regret* function can be understood as the gap between the current selection and the optimal selection. For example, in Fig. 1, client $S_1$ can choose to initiate asymmetric transmission with client $S_3$ and $S_4.$ When selecting $S_4$ as the downlink client, it can obtain a performance gain of 20 Mbps, while selecting $S_3$ as the downlink client, it can only obtain a performance gain of 10 Mbps. Thus, we can say that selecting $S_3$ as the downlink client will lose some performance gain. Accordingly, we define the *Regret* function $L^i_j$ as:

6 $$\begin{aligned} L^i_j = 1 - R^i_j = 1 - log_2(1+c_{ij})/log_2(1+c_{max}). \end{aligned}$$

### Convergence improvement

Convergence is an important property in reinforcement learning. If the convergence is too slow, it indicates that there are more times to explore unknown environments and fewer times to use the optimal policy. In this case, excessive exploration will seriously affect the network performance improvement. If the convergence is too fast, it may lead to insufficient explorations and inability to obtain the optimal policy, resulting in long-term performance loss. Especially in the dynamic wireless communication environment, the learning algorithm needs to quickly obtain the optimal policy by constantly interacting with the environment. In order to improve the speed of convergence while maintaining the correctness of the learning algorithm, we introduce a learning amplification factor (LAF) $\rho$ when updating the posterior probability. Accordingly, the update rules for hyper-parameter $\alpha$ and $\beta$ are:

7 $$\begin{aligned} \alpha (i,j)= & \alpha (i,j) + \rho R^i_j, \end{aligned}$$

8 $$\begin{aligned} \beta (i,j)= & \beta (i,j) + \rho L^i_j, \end{aligned}$$

where $\alpha (i,j)$ and $\beta (i,j)$ are hyper-parameter $\alpha, \beta$ for the asymmetric transmission $S_i \rightarrow AP \rightarrow S_j$.

In Beta function, the expected value $\mu$ and variance $\sigma$ of a Beta random variable can be calculated by:

9 $$\begin{aligned} \mu= & \frac{\alpha }{\alpha + \beta }, \end{aligned}$$

10 $$\begin{aligned} \sigma= & \frac{\alpha \beta }{(\alpha + \beta )^2 (\alpha + \beta + 1)}. \end{aligned}$$

In the full-duplex transmission scheduling algorithm, $\mu$ can be seen as the average *Reward*, which is used to judge whether the scheduled policy is good or not. Variance $\sigma$ represents the confidence in the average *Reward*. The larger the variance, the more uncertain it is and the more times we need to explore.

Next, we theoretically prove that the introduction of LAF will not affect the correctness of policy learning, while also improving the convergence speed of Bayesian optimization.

#### Lemma 1

*The introduction of LAF will not affect the correctness of policy learning.*

#### Proof

In the Beta function, its expected value represents the average *Reward* value, and the scheduler will make decisions based on this expected value. If the introduction of LAF $\rho$ does not change the expected value $\mu,$ we can say that LAF will not affect the correctness of the final optimal policy. This can be demonstrated by comparing the expected values before and after the introduction of LAF. The expectation for using the LAF is denoted by $\mu,$ and the expectation for not using the LAF is expressed as $\mu ',$ then:

11 $$\begin{aligned} \mu = \frac{\rho \alpha }{\rho \alpha + \rho \beta } = \frac{\alpha }{\alpha + \beta } = \mu '. \end{aligned}$$

Therefore, we can say that the introduction of LAF does not affect the correctness of policy learning. $\square$

#### Lemma 2

*The introduction of LAF can improve the convergence speed of the algorithm.*

#### Proof

From the perspective of convergence, a decrease in variance means an increase in the certainty of the expected value, a decrease in the number of explorations, and therefore an increase in the convergence of the algorithm. Here, $\sigma$ represents the variance after introducing the amplification factor, and $\sigma '$ represents the variance before introducing the amplification factor. If $\sigma /\sigma ' < 1,$ it indicates that the variance decreases and the convergence speed increases. If $\sigma /\sigma ' > 1,$ it indicates that the variance increases and the convergence speed decreases. And if $\sigma /\sigma ' = 1,$ it indicates the convergence does not change. The calculation is as follows:

12 $$\begin{aligned} \frac{\sigma }{\sigma '}&= \frac{\frac{\rho \alpha \rho \beta }{(\rho \alpha + \rho \beta )^2(\rho \alpha + \rho \beta + 1)}}{\frac{\alpha \beta }{(\alpha + \beta )^2(\alpha + \beta + 1)}}= \frac{\alpha + \beta + 1}{\rho \alpha + \rho \beta + 1}. \end{aligned}$$

Considering that $\alpha$ and $\beta$ are both positive numbers, if we set $\rho >1,$ ${\sigma }/{\sigma '} < 1,$ the convergence speed will be increased. $\square$

### Dynamic environment support

In Bayesian optimization algorithms, as the number of explorations increases, the Beta distribution tends to stabilize and the variance decreases. At this time, the scheduler will exploit more and explore less. When the communication environment remains stable, this characteristic can steadily improve the overall performance of the system after exploring the optimal policy. However, wireless communication environments are usually non-stationary. Therefore, it is necessary to maintain the sensitivity of the algorithm to the environment and quickly adjust the optimization parameters when the communication environment changes. To support the dynamic communication environment, the variance of the Beta distribution should not be too small from a mathematical perspective. Therefore, we design a window-constraint parameter adjustment method, which increases the variance value while not affecting the correctness of policy learning. We set a specific window-constraint value $\varpi .$ When $\alpha + \beta > \varpi,$ we will scale down these two parameters $\alpha$ and $\beta$:

13 $$\begin{aligned} \alpha= & \frac{\varpi }{\alpha + \beta } \alpha, \end{aligned}$$

14 $$\begin{aligned} \beta= & \frac{\varpi }{\alpha + \beta } \beta . \end{aligned}$$

Here, the scaling factor is denoted as $\varrho = \frac{\varpi }{\alpha + \beta }$ $(0<\varrho <1).$ Then, the expected value and variance based on the window-constraint method can be calculated by:

15 $$\begin{aligned} \mu= & \frac{\varrho \alpha }{\varrho \alpha + \varrho \beta } = \frac{\alpha }{\alpha + \beta }, \end{aligned}$$

16 $$\begin{aligned} \sigma= & \frac{\varrho \alpha \varrho \beta }{(\varrho \alpha + \varrho \beta )^2 (\varrho \alpha + \varrho \beta + 1)}=\frac{\alpha \beta }{(\alpha + \beta )^2 (\varrho \alpha + \varrho \beta + 1)}. \end{aligned}$$

From (15) and (16), we can see that the expected value $\mu$ remains unchanged before and after scaling, which means that the correctness of policy learning will not be affected. And for the variance, the value increases since $\varrho < 1.$ Therefore, we can say that the window-constraint adjustment method can improve adaptability in dynamic communication environments and ensure the correctness of policy learning.

### Algorithm description

The proposed WCB algorithm description is presented in Algorithm 1. One input to the Algorithm is the client number. It should be noted that the input client number needs to indicate whether the client is an uplink or downlink client number. For the convenience of description, we use $S_i$ and $S_j$ to represent the uplink client and downlink client, respectively. The other inputs are *Reward* and *Regret*. These two values are delay inputs that are obtained after frames are transmitted according to the optimized policies. The output is the optimized policy, which is a client number in the asymmetric transmission corresponding to the input client number. For example, if the input is an uplink client number, the output will be a downlink client number.

**Algorithm 1:**
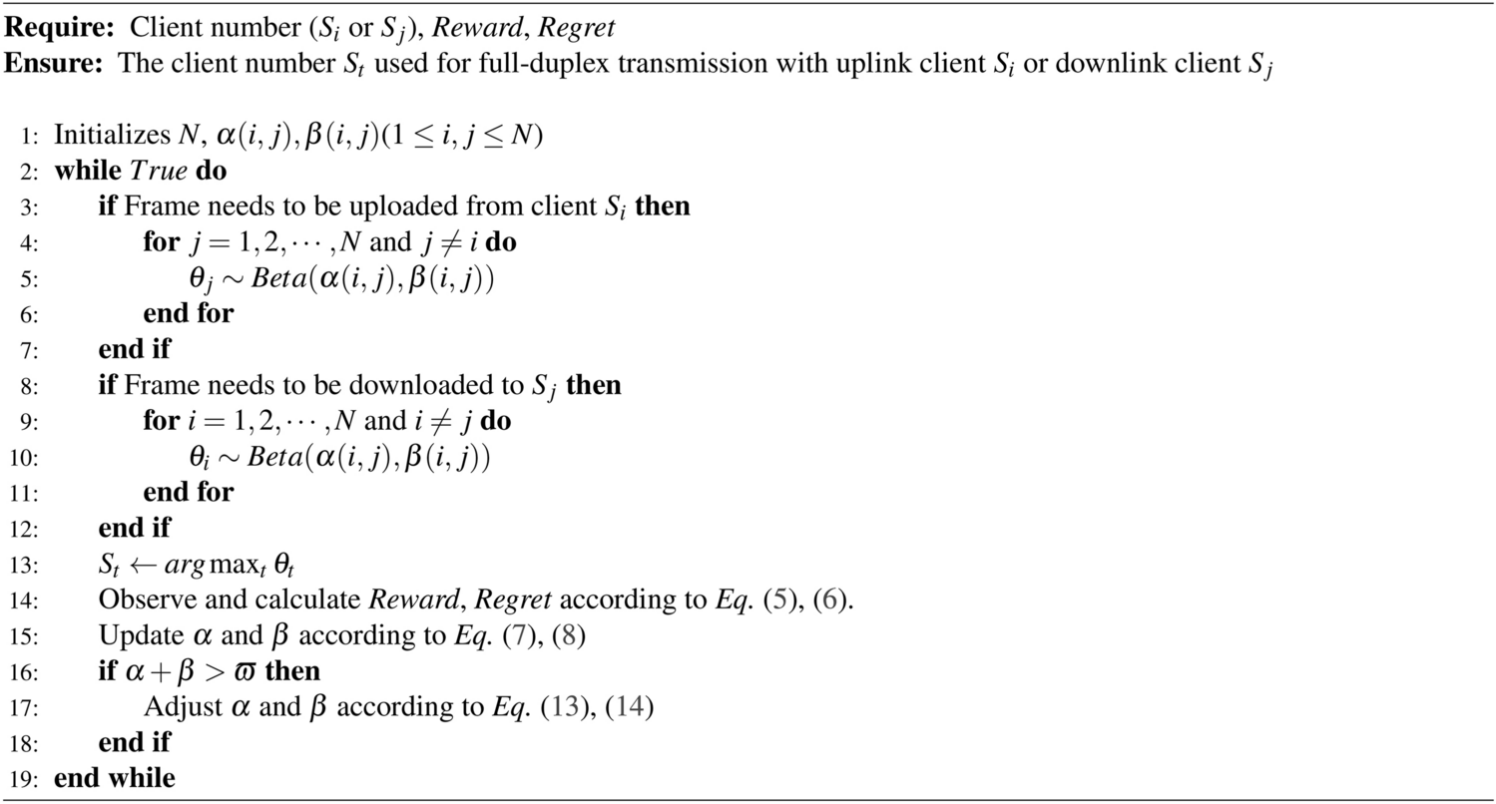
The Window-Constraint Bayesian algorithm

Before the algorithm starts running, it is necessary to initialize the parameters used in the algorithm. Variable *N* is initialized according to the number of clients registered in the network. Considering that the uplink client has $N-1$ downlink client to choose from, we use two matrices to identify the hyperparameter to be used: $\varvec{\alpha }=(\alpha (i,j))_{N \times N}$ and $\varvec{\beta }=(\beta (i,j))_{N \times N}.$ And $\alpha (i,j)$ and $\beta (i,j)$ are used to characterize the *Reward* distribution of asymmetric transmission $S_i \rightarrow AP \rightarrow S_j.$ The hyperparameter $\alpha (i,i)$ and $\beta (i,i)$ are not involved in policy learning, since it is assumed that clients do not support full-duplex mode. If some clients enable the full-duplex mode, we only need to add the corresponding hyperparameter $\alpha (i,i)$ and $\beta (i,i)$ to the policy learning, then the algorithm will also support full-duplex nodes. All hyperparameters in $\varvec{\alpha }$ and $\varvec{\beta }$ are initialized to 1.

Then, the algorithm will enter a continuous updating iteration. If the input is the uplink client number $S_i,$ we need to search for an optimal downlink client $S_j.$ Thus, we will sample a group of expected value ${\theta _j}$ using the Beta function with $\alpha (i,j),\beta (i,j)$ $(1 \le j \le N, j \ne i).$ And if the input is the downlink client number $S_j,$ we need to search for an optimal uplink client $S_i.$ We will also sample a group of expected value ${\theta _i}$ using the Beta function with $\alpha (i,j),\beta (i,j)$ $(1 \le i \le N, i \ne j).$ Whether choosing the uplink client or the downlink client, the optimal transmission policy is to select the client number corresponding to the maximum sampling value. After the transmission according to the optimized policy is over, we can observe and calculate the *Reward* and *Regret* values. And the parameters $\alpha,\beta$ will be updated accordingly. And if $\alpha + \beta > \varpi,$ we should adjust these two parameters according to (13) and (14). At this point, the algorithm execution ends. It will wait for the next input to enter the next loop.

## Two-way handshake transmission mechanism

With the CSMA mechanism, wireless nodes compete for the wireless channel in a distributed manner. To utilize symmetric transmissions, there will be two cases: (1) a client first preempts the wireless channel to upload data, and then the AP needs to select a downlink client; (2) the AP first preempts the wireless channel to initiate downlink transmission, and at this time, the AP also needs to notify a client to start an uplink transmission. In addition, considering that the uplink signal may affect the downlink signal in asymmetric transmissions, we should design an effective mechanism to obtain the SINR value of the downlink transmission, so that the AP can choose an appropriate downlink transmission rate. The details of the transmission procedure are introduced as follows.

**Fig. 3:**
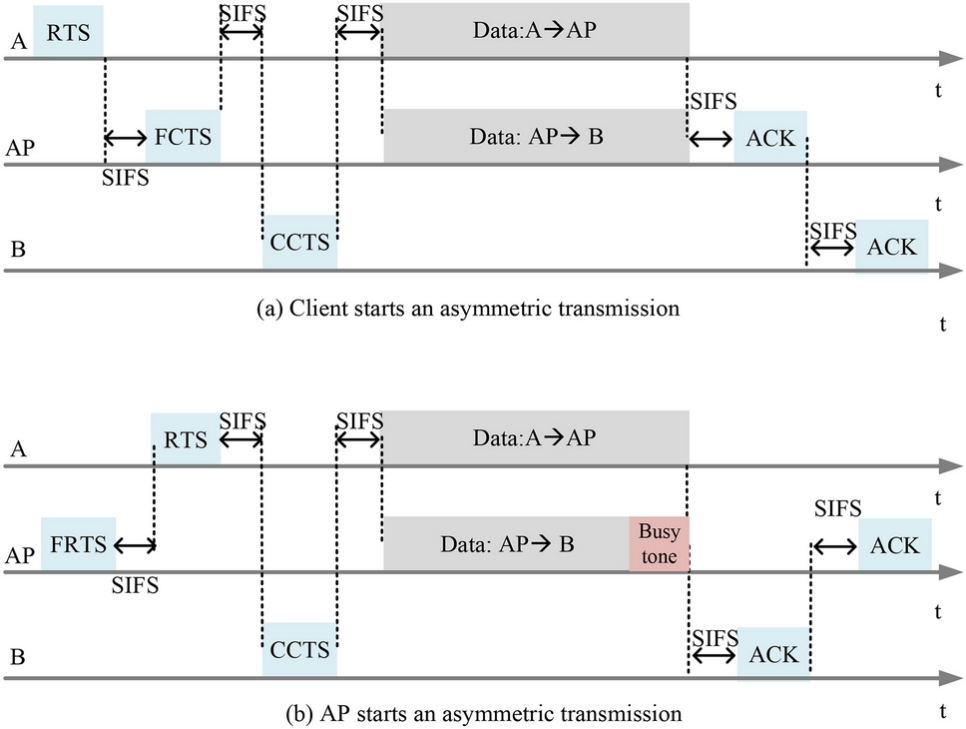
The transmission procedure of RLFD: (**a**) a client firstly preempts the wireless channel and starts an asymmetric transmission. (**b**) The AP first preempts the wireless channel and starts an asymmetric transmission.

### A client first preempts the wireless channel

**Fig. 4:**
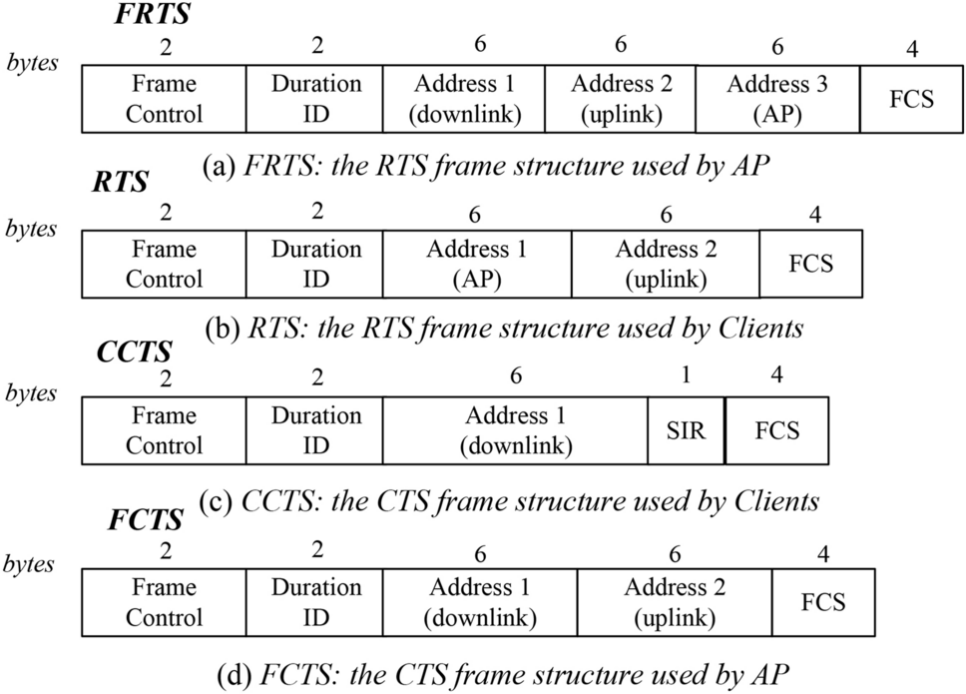
Key frame structures used in RLFD.

As shown in Fig. 3a, when client *A* firstly preempts the wireless channel, it will send a control frame RTS to the AP to request to send. After receiving the RTS frame, AP will reply to client *A* with a Full-duplex Clear-To-Send (FCTS) control frame. Unlike in traditional half duplex wireless networks, the control frame FCTS not only contains the information of “CTS” returned to client *A*, but also the “RTS” request information to client *B*. The frame structures used in RLFD are shown in Fig. 4. When client *B* receives the FCTS frame from the AP, it replies with a Client Clear-To-Send (CCTS) frame, which includes the SINR value of the downlink signal received in this asymmetric transmission. With the SINR value, the AP can select an appropriate data rate for downlink transmission. As for the uplink data rate from client *A* to AP, it can also be determined by the RSSI value measured during the handshake procedure. After the data frame transmission is completed, AP and client *B* will return an Acknowledgment (ACK) frame to confirm whether these frames have been correctly transmitted. It should be noted that when client *B* replies to the ACK confirmation frame, it may affect the transmission of the ACK confirmation frame that AP replies to client *A*. Therefore, when sending the ACK frame, we send them one by one. And we follow the principle of the initiator first reply for ACK in the asymmetric transmission.

### The AP first preempts the wireless channel

As shown in Fig. 3b, when the AP first preempts the wireless channel, it will select a client for uplink transmission according to the downlink transmission. Thus, a full-duplex request-to-send (FRTS) control frame initiated by the AP contains both uplink and downlink address information. When uplink client *A* receives the FRTS frame, it will reply with a RTS frame. For two reasons: (a) the RTS frame will inform its neighbors of the channel occupying time to solve the hidden terminal problem. (b) in order to facilitate the downlink client to hear the uplink signal and calculate the SINR value in the downlink client in the asymmetric transmission. When the AP receives the RTS frame, it will be processed as a confirmation signal and no “CTS” information will be returned. When client *B* receives the FRTS frame, it will return a CCTS frame. Similarly, frame CCTS contains the SINR value of the received downlink signal in the asymmetric transmission. Note that, client *B* should wait for a specific time to return CCTS, in which the specific time equals the time to send the RTS frame plus a short interval frame space (SIFS). After the transmission of uplink/downlink data frame ends, the AP and client will also return ACK frames in sequence.

In addition, the transmission end time of uplink/downlink data frame may be different. If the uplink transmission ends before the downlink transmission, the clients in the network can still sense that the wireless channel is busy, and there will be no hidden terminal problem. But if the downlink transmission ends before the uplink transmission, signal conflicts may occur at the AP. For example, when the downlink transmission ends first, some clients cannot perceive the signal from client *A*. These clients will consider that the channel is idle and start sending RTS frames, which will cause conflicts since client *A* is still uploading data. Besides, if client *B* cannot perceive the signal from client *A*, the timing for client *B* to reply to the ACK frame cannot be determined. In asymmetric transmissions initiated by clients, we can use the network allocation vector (NAV) value to reserve the channel occupying time by specifying the “duration ID” in the control frame, as shown in Fig. 4d. However, in asymmetric transmissions initiated by the AP, the uplink transmission end time cannot be predicted. Here, we use a busy tone to solve the problem caused by inconsistent transmission end times between the uplink and downlink. When the downlink data frame ends before the uplink data frame, the AP will continue to send a busy tone signal until the transmission of uplink data frame is over, as shown in Fig. 3b.

### The calculation of SINR value

In the design of asymmetric transmission, we ensure that the downlink client has the opportunity to hear the transmitting signal from the uplink client. And the received signal strength can be measured during the handshake procedure as well. Therefore, the SINR value for the downlink transmission in an asymmetric transmission can be calculated when the handshake is over. For asymmetric transmission $S_i \rightarrow AP \rightarrow S_j,$ the uplink signal heard by the downlink client can be expressed as *Pr*(*i*, *j*), and the signal strength obtained from the AP is *Pr*(0, *j*). At this point, the SINR value can be calculated according to Eq. (2).

## Performance analysis

This section will analyze the performance of the RLFD protocol with saturated traffic, in which the saturated traffic means that there always have packets to be transmitted between AP and clients. Here, we adopt a Markov chain model to characterize the transmission procedure of RLFD, which has been successfully applied in the performance analysis of IEEE 802.11 DCF^32^.

### Full-duplex transmission probability

Similar to^32^, we split the continuous time into a small time slot, and each node in the wireless network competes for the wireless channel at the beginning of the time slot. Then, the transmission probability $P_{tx}$ for a client in a slot can be calculated as:

17 $$\begin{aligned} P_{tx} = \frac{2(1-2p)}{(1-2p)(W+1) + pW(1-(2p)^m)}, \end{aligned}$$

in which, *W* represents the minimum contention window value in the CSMA/CA mechanism, and $2^m W$ is the maximum contention window value. In real wireless communication systems, to maintain transmission fairness and optimize the network performance, AP and clients may set up different contention windows. For example, AP can set up a smaller contention window to obtain more opportunities to start a transmission. Here, the minimum/maximum contention window for AP is expressed as $W_a,2^{m_a} W_a.$ *p* is the conditional conflict probability, which represents the probability that two or more nodes in the network try to compete with the channel at the same time. And conflict may occur between AP and clients, or two or more clients. The conditional conflict probability can be calculated by:

18 $$\begin{aligned} p = 1 - (1 - P_{tx})^{N-1}, \end{aligned}$$

in which, *N* is the number of clients in the wireless network. By solving (17) and (18), we can get the value of *p* and $P_{tx}$.

Similarly, we can also obtain the transmission probability $P^a_{tx}$ and the conditional conflict probability $p_a$ for the AP, where the transmission probability $P^a_{tx}$ can be expressed as:

19 $$\begin{aligned} P^a_{tx} = \frac{2(1-2p_a)}{(1-2p_a)(W_a+1) + pW_a(1-(2p_a)^{m_a})}, \end{aligned}$$

the conditional conflict probability $p_a$ can be calculated as:

20 $$\begin{aligned} p_a = 1 - (1 - P^a_{tx})^{N}. \end{aligned}$$

By solving (19) and (20), we can get the value of $p_a$ and $P^a_{tx}$.

Considering that there is one AP and N clients in the network, the probability of at least one node initiating a transmission in the network is expressed as $P_{tr}$:

21 $$\begin{aligned} P_{tr} = 1 - (1-P^a_{tx})(1-P_{tx})^N. \end{aligned}$$

And the probability of only the AP attempting to initiate a transmission is expressed as $P_{ap}$:

22 $$\begin{aligned} P_{ap} = P^a_{tx}(1-P_{tx})^N. \end{aligned}$$

When only one client tries to compete for the channel, since AP operates in full-duplex mode, it can always hear the contention signals regardless of whether AP participates in the channel preemption. Thus, the probability that only one client obtains the channel $P_{client}$ can be calculated as:

23 $$\begin{aligned} P_{client} = N\cdot P_{tx}\cdot (1-P_{tx})^{N-1}. \end{aligned}$$

Then, the conflict probability $P_c$ of two or more nodes attempting to initiate transmission simultaneously in the network can be calculated as:

24 $$\begin{aligned} P_c = P_{tr} - P_{ap} - P_{client} = 1 - (1 + (N-1)P_{tx})(1-P_{tx})^{N-1}. \end{aligned}$$

### Efficient full-duplex payload

In asymmetric transmission, the efficient full-duplex payload includes uplink data frames from clients to the AP and downlink data frames from the AP to clients. Here, $L_{Data_1}$ and $L_{Data_2}$ are used to represent the uplink data frame length and the downlink data frame length, respectively. Without loss of generality, we use transmission time to characterize the frame length directly here. The frame transmission time $T_{frame}$ can be calculated as:

25 $$\begin{aligned} T_{frame} = L_{frame}/r_t, \end{aligned}$$

in which, $L_{frame}$ means the frame length and $r_t$ is the transmission rate. According to (25), the transmission times of control frames such as FRTS, RTS, RCTS, CCTS, and ACK are expressed as $T_{FRTS},$ $T_{RTS},$ $T_{FCTS},$ $T_{CCTS},$ $T_{ACK}$; The transmission time of uplink and downlink data frames are $T_{Data_1}$ and $T_{Data_2},$ respectively.

Since we use the handshake mechanism to start transmission, we lose a RTS/FRTS frame when conflict happens. Specifically, when two or more clients try to start transmission at the same time, we lose a transmission time $T^{rts}_c$:

26 $$\begin{aligned} T^{rts}_{c} = T_{RTS} + T_{DTFS}, \end{aligned}$$

when the AP tries to start a transmission and conflict happens, the losing time is $T^{frts}_c$:

27 $$\begin{aligned} T^{frts}_{c} = T_{FRTS} + T_{DTFS}. \end{aligned}$$

When the client first preempts the channel and initiates an asymmetric transmission, as shown in Fig. 3a, the successful transmission time $T^{rts}_{s_1}$ will be:

28 $$\begin{aligned} T^{rts}_{s_1} = T_{RTS} + T_{FCTS} + T_{CCTS} + T_{Data} + 2 T_{ACK} + 5 T_{SIFS} + T_{DIFS}. \end{aligned}$$

And when the AP first preempts the channel and initiates an asymmetric transmission, as shown in Fig. 3b, the successful transmission time $T^{rts}_{s_2}$ will be:

29 $$\begin{aligned} T^{rts}_{s_2} = T_{FRTS} + T_{RTS} + T_{CCTS} + T_{Data} + 2 T_{ACK} + 5 T_{SIFS} + T_{DIFS}, \end{aligned}$$

where $T_{DIFS}$ represents a DCF inter-frame space-time and $T_{SIFS}$ represents a short inter-frame space-time. $T_{Data} = \max \{T_{Data_1},T_{Data_2}\}$.

The efficient full-duplex payload of an asymmetric transmission is the sum of the uplink data frame and the downlink data frame, that is, $T_{Data_1}+T_{Data_2}.$ And we define the payload ratio $\eta,$ which means the ratio of improvement in full-duplex mode compared to half-duplex mode:

30 $$\begin{aligned} \eta = \frac{T_{Data_1} + T_{Data_2}}{T_{Data}} = \frac{1 + \frac{r_1}{r_2}\frac{L_{Data_2}}{L_{Data_1}}}{\max \{1, \frac{r_1}{r_2}\frac{L_{Data_2}}{L_{Data_1}} \}}, \end{aligned}$$

where $r_1,r_2$ represent uplink and downlink data rates, respectively. According to the definition of $\eta,$ it is easy to obtain: $1 \le \eta \le 2 .$ And when $\eta =1,$ it indicates that there is no full-duplex transmission opportunity, while when $\eta =2,$ it indicates that the full-duplex transmission opportunity has been fully utilized. Besides, as shown in Eq. (30), to optimize the payload ratio, both *Frame length* and *data rate* should be considered simultaneously.

### Saturation network throughput

With the probability of successful transmission in each time slot and the transmission time of different frames, the network throughput *S* is defined as the ratio of the successfully transmitted data frames length to the total transmission time over a period of time, which can be calculated as:

31 $$\begin{aligned} S = \frac{(P_{ap}+ P_{client}) \eta T_{Data}}{(1-P_{tr})T_{slot} + P_{client}T^{rts}_{s_1} + P_{ap}T^{rts}_{s_2} + P_c T^{rts}_{c}}, \end{aligned}$$

where $T_{slot}$ is the time for a slot.

From (31), we can see that there is a linear relationship between network throughput and payload ratio. The larger the payload ratio, the greater the network throughput. In other words, the larger the payload ratio, the more full-duplex opportunities will be exploited. Therefore, optimizing the payload ratio is to optimize the throughput of the full-duplex network.

## Performance evaluation

In this section, numerical and simulation results are both presented to evaluate the performance of the RLFD MAC protocol. Firstly, we compare the network throughput of RLFD MAC protocol with traditional IEEE 802.11 DCF protocol and study the impact of the contention window on the performance of our proposed protocol based on the analytical model. Then, we develop a simulation program in Python and validate the effectiveness of the designed WCB algorithm and compared its performance with the classic $\epsilon -$greedy algorithm and random selection algorithm. Thirdly, we tested the network throughput and investigated how LAF and window-constraint value in the WCB algorithm affect the network performance with the simulation program as well.

### Simulation setup

#### Topology and traffic model

We assume that there is one AP and several clients in the full-duplex wireless network used for simulation. All network nodes are deployed in a rectangular area of 100 m $\times$ 100 m, clients are randomly distributed within the area, and the AP is located at the center of the rectangular area. The default number of clients in the network is set to $N=40.$ To ensure the credibility of test data and reduce the error of randomly generated topology, 100 random topologies are generated for each test and the average of the test values is taken. As for the network traffic model, saturated traffic is considered in this paper, which means that the AP always has packets to clients, and clients always have packets to the AP. And the frame length settings are shown in Table 1.

**Table 1:** Parameters for PHY and MAC layers.

Parameter	Value	Parameter	Value
PHY header	16 bytes	FRTS	26 bytes
MAC header	34 bytes	FCTS	20 bytes
ACK	14 bytes	CCTS	15 bytes
RTS	20 bytes	Slot	20 us
CTS	14 bytes	SIFS	10 us
Payload	1500 bytes	DIFS	50 us

**Table 2:** Data rates and SNR under different packet loss rates.

Modulation	Coding rate	Data rate (Mbps)	$SNR_t$ (dB)	$SNR_u$ (dB)
BPSK	1/2	6	5	9.3
BPSK	3/4	9	6	10.3
QPSK	1/2	12	7	11.3
QPSK	3/4	18	9	13.3
16-QAM	1/2	24	13	17.3
16-QAM	3/4	36	17	21.3
64-QAM	1/2	48	20	24.3
64-QAM	3/4	54	22	26.3

#### Physical and MAC model

The control frame and data frame are similar to that in IEEE 802.11 DCF, and some extensions are made for full-duplex communication, such as FRTS, CCTS, as shown in Fig. 4. The parameters for PHY and MAC layer are selected according to Table 1. To simplify the calculation of SNR/SINR values, we use a log distance propagation loss model, which can be derived from (1):

32 $$\begin{aligned} L(r) = L(r_0) + 10 n log_{10}(r/r_0) [dB], \end{aligned}$$

in which *r* is the propagation distance and *n* is the pass loss exponent. And we set $n=4$ to reflect the indoor environment^33^. $r_0$ is a reference distance and $L(r_0)$ is the propagation loss value at $r_0,$ which is set to $r_0 = 1$ m and $L(r_0) = 30$ dB, respectively. In addition, the transmission power of the AP and clients is set to 50 dbm and 47 dbm, respectively. The background noise in the communication environment is − 70 dbm, while the sensitivity of wireless devices to perceive wireless signals is − 85 dbm.

#### Multi-rate transmission model

Assumed that all wireless nodes support multi-rate transmission and have different packet loss rates at different data rates under different SNR conditions. Generally speaking, wireless devices will choose different modulation modes and encoding rates in different wireless communication environments, thereby obtaining different data rates. The relationship between the data rate and SNR value used in the simulation is shown in Table 2. There are four modulation methods, two encoding rates, and eight corresponding data rates to choose from. Note that there are two SNR thresholds, in which $SNR_t$ and $SNR_u$ represent the recommended threshold and the optimal threshold, and the corresponding packet loss rate is set to $5\%$ and 0, respectively.

### Results

#### Performance results based on the protocol model

To validate the effectiveness of the proposed RLFD protocol, we first evaluate the network throughput based on the analytical model under saturated traffic. We set the contention window to 64 ($W=64$) and the maximum contention window to 2048 ($m=5$) for both the AP and clients. For simplicity, we set the data rate to 1 Mbps. The other parameters in the protocol model are selected according to Table 1. As for payload ratio $\eta,$ as shown in Eq. (31), it can largely affect the performance of the protocol and it is mainly determined by the scheduling algorithm. Here, we provide the best-case ($\eta = 2$) and worst-case ($\eta = 1$) network throughput of the RLFD protocol and compare its performance with that of the IEEE 802.11 DCF with and without RTS/CTS mechanism.

**Fig. 5:**
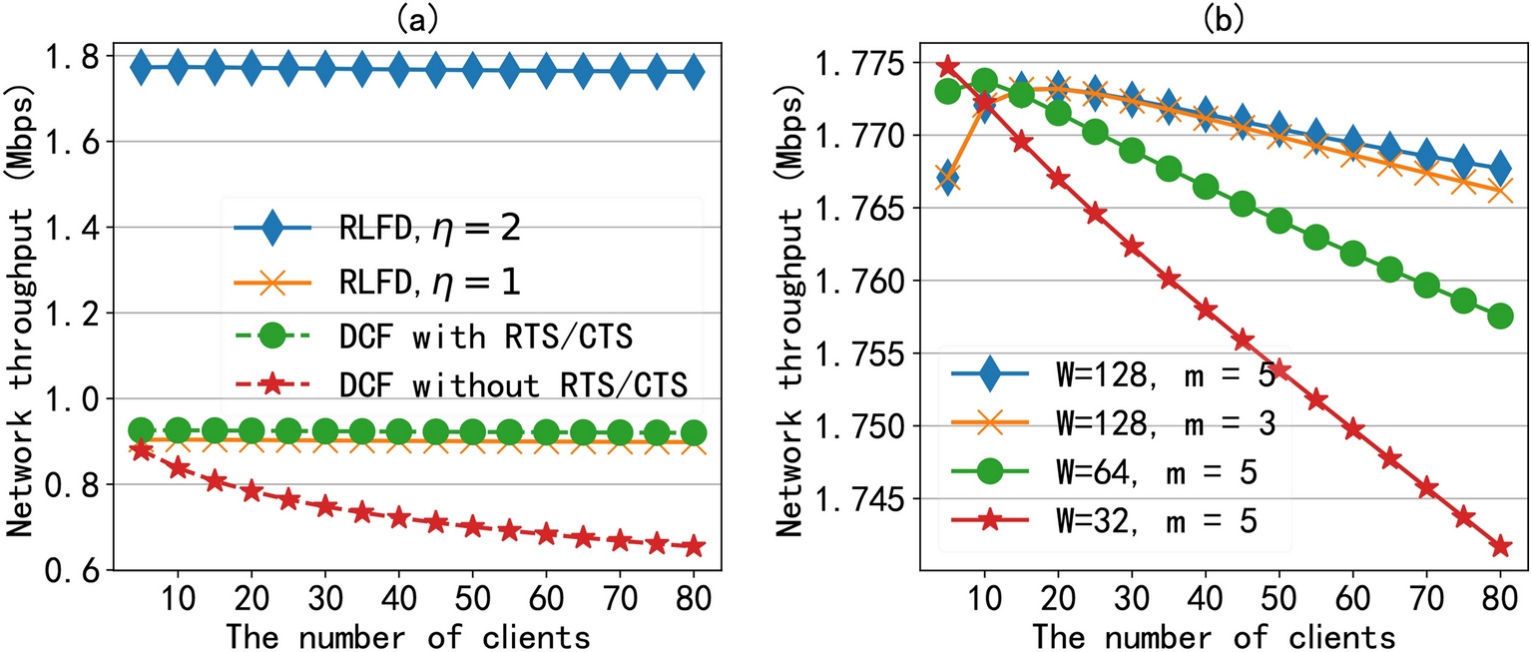
The network throughput under protocol model: (**a**) the network throughput of different MAC protocols. (**b**) The network throughput of RLFD with different contention windows.

As shown in Fig. 5a, when $\eta = 1,$ it means that there is no full-duplex transmission opportunity available in the network, and the performance of the RLFD protocol is the worst. At this time, the network throughput of RLFD will be slightly worse than that of IEEE 802.11 DCF with RTS/CTS mechanism in half-duplex mode, as RLFD introduces an additional handshake negotiation process. However, since our proposed protocol uses the handshake mechanism, compared to the IEEE 802.11 DCF without RTS/CTS mechanism, it can maintain good performance when the network size increases. On the other hand, when there are many asymmetric transmissions in the network and the quality of selectable downlink signals is good, it can be assumed that the RLFD protocol achieves best-case network performance ($\eta = 2$), which can achieve nearly twice the network throughput compared to the IEEE 802.11 DCF with RTS/CTS. In a real full-duplex network, the value of payload ratio $\eta$ is between 1 and 2, so the actual network throughput will also be between the best-case performance and worst-case performance. Due to the difficulty in obtaining the payload ratio through calculation, the performance results of the RLFD protocol under saturated traffic will be verified by simulations. And in the next subsection, we can see that the average payload ratio $\eta \approx 1.92$ when the number of clients is set to 40.

In addition, we also calculated and analyzed the impact of the key parameter contention window in the protocol on network performance. As shown in Fig. 5b, we compared the protocol performance under four different contention window values. When the minimum contention value $W=32$ and the maximum contention window value is 1024 ($m=5$), the network throughput decreases as the network size increases. The reason is that the increase in clients number leads to increased competition between clients, but a small contention window increases the probability of collisions, resulting in bad performance. For the other three groups with large contention windows, the network throughput first increases and then decreases with the increase of clients number. The reason is that when there are fewer network nodes, an excessive contention window can lead to longer channel vacancy time, resulting in performance loss. As the number of network nodes increases, channel utilization opportunities increase, resulting in an upward trend in performance. However, as the number of nodes continues to increase, the probability of node conflicts increases, leading to an increase in network performance loss. Therefore, in a real full-duplex wireless network, the contention window should be set based on the network size. If there are a large number of clients, a large contention window should be used.

#### Efficiency of the proposed WCB algorithm

To demonstrate the effectiveness of the proposed WCB algorithm, two typical scheduling strategies are compared in the simulation. One is the $\epsilon -$greedy algorithm, in which the AP exploits the observed optimal choice with probability $1-\epsilon$ and randomly explores with probability $\epsilon .$ And we set $\epsilon = 0.1.$ Another approach is a random selection strategy, in which we assume that the AP randomly selects the downlink client in an asymmetric transmission with the same probability. For the proposed WCB algorithm, we set the LAF $\rho = 50$ and window-constraint value $\varpi = 500.$ The number of clients is set to 40. The other simulation parameters are selected according to Table 1 and 2. And two typical metrics are used to test the proposed algorithm, that is, average reward and optimal action. The average *Reward* value is the performance gain in full-duplex transmissions and can be calculated by Eq. (5). The optimal action is the probability of selecting the best downlink in an asymmetric transmission. These two metrics can directly evaluate the quality of the second link in the asymmetric transmission selected by a scheduling algorithm, thus affecting the throughput of the full-duplex network.

**Fig. 6:**
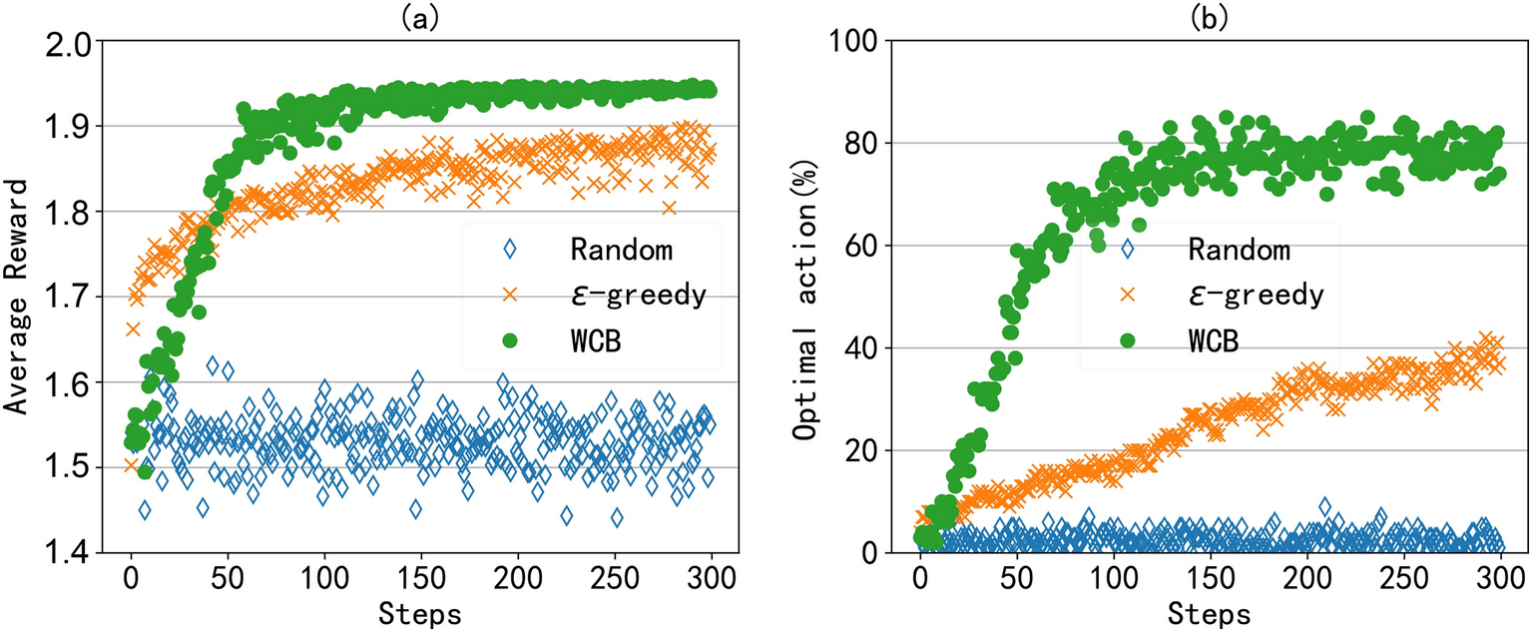
Efficiency of the WCB algorithm in RLFD: (**a**) the average reward with different algorithms. (**b**) The optimal action with a different algorithm.

**Fig. 7:**
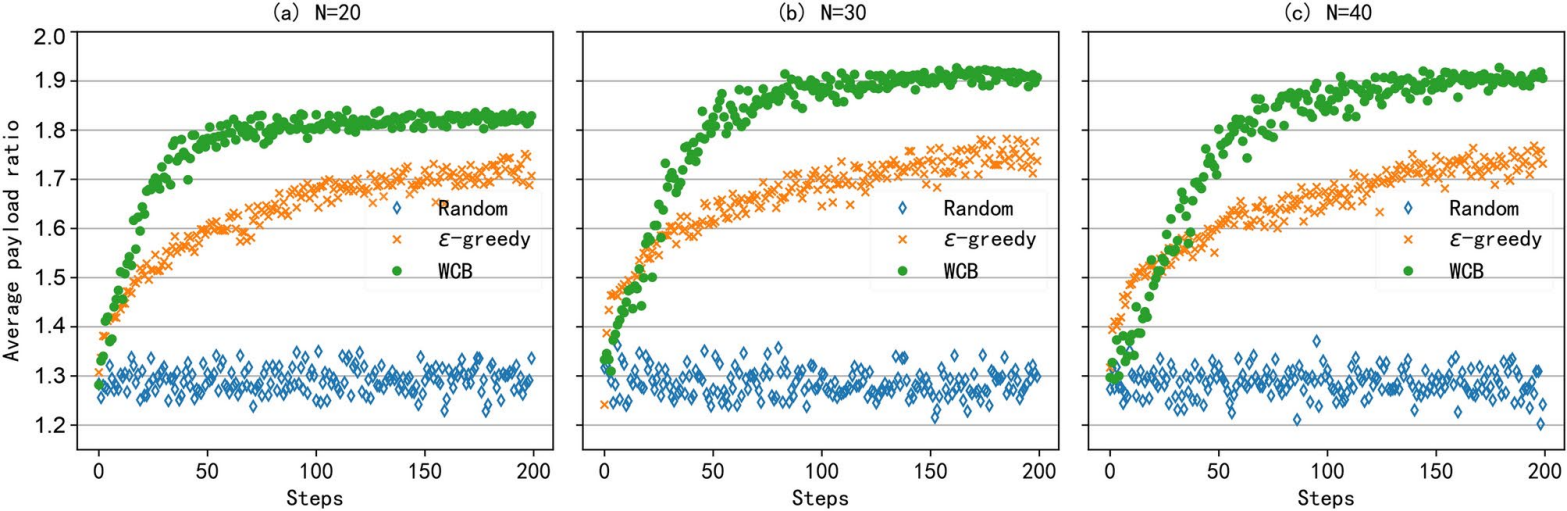
The average payload ratio with different scheduling algorithms and different numbers of clients.

We first tested the average *Reward* values returned by three algorithms. As shown in Fig. 6a, among the three algorithms, the proposed WCB algorithm can obtain the best *Reward* value and achieve a fast convergence. Compared to the random selection strategy, the WCB algorithm can improve the average *Reward* value by $(0.92 - 0.52)/0.52=77\%.$ And compared with $\epsilon$-greedy algorithm can improve the average *Reward* value by $(0.92 - 0.88)/0.88=4.5\%.$ Correspondingly, we also test the probability of optimal action by selecting the best downlink in an asymmetric transmission with the three algorithms, as shown in Fig. 6b. The WCB algorithm can obtain the optimal probability of about 80%, $\epsilon$-greedy algorithm can achieve the optimal action with a probability of about $40\%.$ And the convergence of the $\epsilon$-greedy algorithm is slower than that of the WCB algorithm. In the random selection strategy, the probability of selecting the best downlink is about $1/40 = 2.5\%.$ Moreover, by comparing Fig. 6a,b, although the probability of the optimal action for $\epsilon$-greedy algorithm is only half of that of the proposed WCB algorithm, its average reward is near that of WCB, indicating that $\epsilon$-greedy algorithm may select a sub-optimal downlink client in many asymmetric transmissions.

Then, considering that payload ratio ($\eta$) can reflect the utilization of full-duplex opportunities and determine network throughput, as shown in Eq. (31), we test the average payload ratio under different network sizes. Three different network sizes were selected, namely 20, 30, and 40 clients in the network. The results are shown in Fig. 7, and it can be seen that RLFD with the WCB algorithm can achieve the best performance under different network sizes. And compared to the $\epsilon$-greedy algorithm, the WCB algorithm converges faster. However, the convergence varies in the three different network sizes. As the network size increases, the AP needs a longer time to explore and learn the optimal policy, resulting in a decrease in convergence speed. And as the network size increases, the throughput improvement brought by the downlink also increases. The reason is that as the number of clients in the network increases, the full-duplex opportunity increases, resulting in improved network performance. Of course, this throughput improvement reaches its maximum when the AP already has a great chance of initiating the best asymmetric transmission. As shown in Fig. 7b,c, the average downlink throughput for the number of clients $N=30$ and $N=40$ are basically the same.

**Fig. 8:**
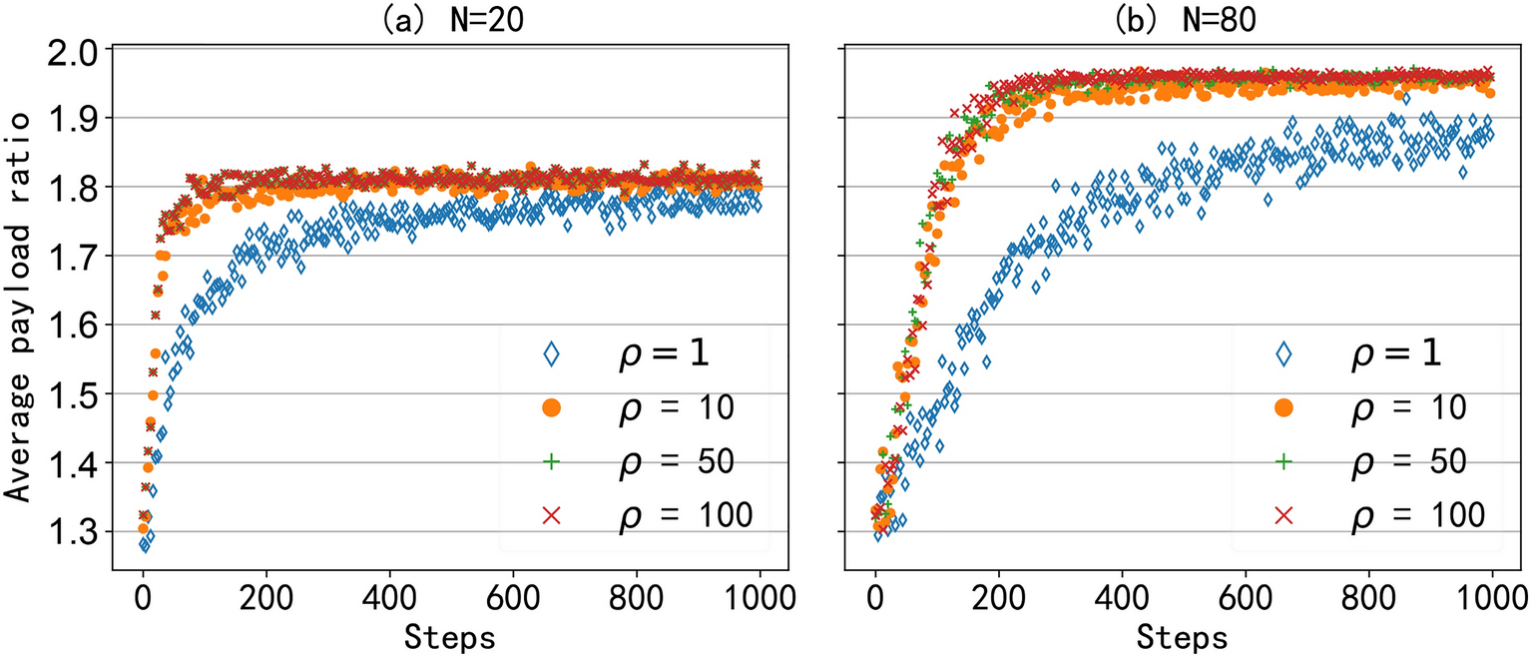
The average payload ratio with different LAF values and different numbers of clients.

#### Convergence

To evaluate the convergence of the WCB algorithm, we conduct a test with different LAF values and different numbers of clients. In the simulation, the window-constraint value is set to 500. Four different LAF values are selected, namely $\rho = 1, 10, 50, 100.$ As shown in Fig. 8, as the LAF value increases, the convergence speed increases and all converge to the same value. Thus, we can say that the introduction of LAF can improve the convergence speed while maintaining the correctness of the WCB algorithm. Of course, the improvement of convergence speed by increasing the LAF value is limited. On one hand, we can see that when the value of LAF increases from 10 to 100, the convergence speed nearly does not increase. For a specific network size, there is an upper limit for convergence speed, and this upper limit cannot be increased by increasing the value of LAF. On the other hand, by comparing Fig. 8a,b, we can see that the larger the network size, the slower the convergence speed of the algorithm. The reason is that each asymmetric transmission needs to be explored at least once before we exploit the optimal policy. And when the network size is large, there are more full-duplex opportunities, and the gap between optimal and sub-optimal is also small, requiring more learning steps to converge. Thus, when the network size is large, even if the algorithm does not converge, the network may still obtain better performance than that of small network size. For example, when the AP learns 200 steps, the payload ratio with 20 clients has converged to its maximum value, and the network with 80 clients has not yet converged. But the payload ratio of 80 networks has already exceeded the payload ratio of 20 networks at this time.

**Fig. 9:**
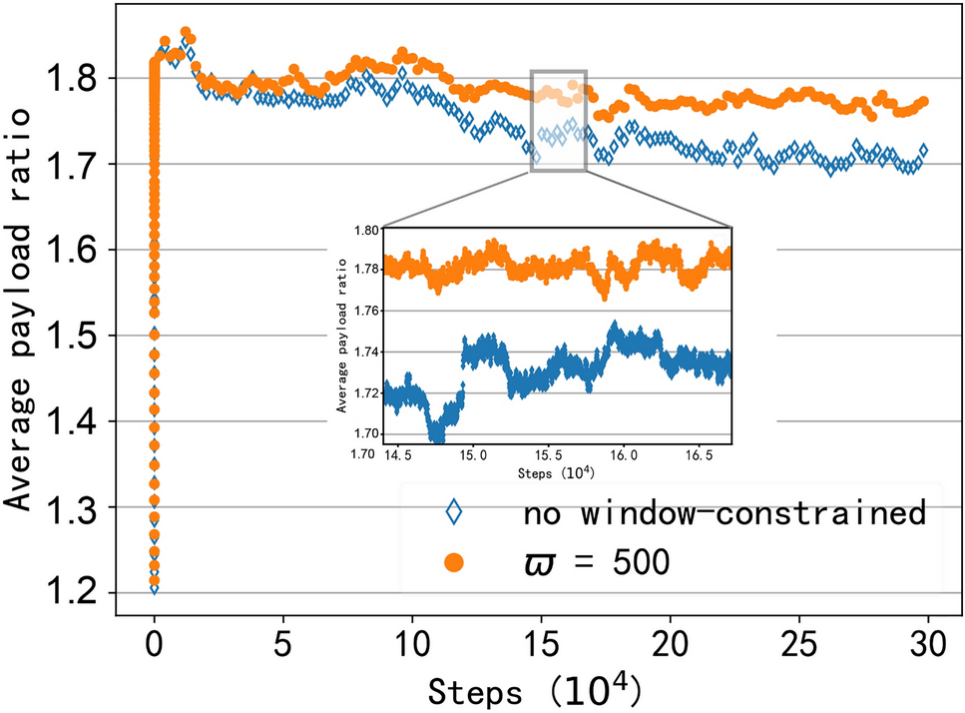
The average payload ratio under dynamic communication environment.

#### Dynamic communication environment support

To simulate the dynamic environments, two aspects have been considered. On the one hand, we assume that nodes are constantly moving and use a random walk model as the moving model. Clients randomly choose a direction and move at a speed of 5 m/s. On the other hand, due to the susceptibility of wireless signals to interference, the received signal strength will be affected by unknown noise. Thus, we added noise to the received signal, and the noise follows a Gaussian distribution with the standard deviation is 0.1 times the received signal strength. For example, if the received signal strength is 30 db, the standard deviation is 3. Besides, we assume that there are 20 clients in the wireless network. The window-constraint value and LAF are set as $\varpi = 500, \rho = 50.$ We compared the impact of WCB algorithms with and without window constraints on network performance, and the average payload ratio under the dynamic environment has been evaluated. As shown in Fig. 9, when the WCB algorithm uses the window-constraint method, we can see that the average payload ratio remains steady, which means our proposed algorithm can obtain a stable network performance in dynamic environments. However, if the window-constrained method is not applied in WCB, the network performance shows a declining trend. This is because, in the long-term running process, the super-parameter value ($\alpha$ and $\beta$) in the beta function becomes too large, resulting in a slow convergence. Besides, by zooming in on the details in Fig. 9, we can see that the network performance exhibits a “jagged” pattern. This is because the movement of clients will cause changes in the network topology and communication environment, and the AP needs to re-learn to recover to better network performance. This jagged change also indicates that the RLFD protocol can quickly converge and achieve good network throughput in dynamic communication environments.

**Fig. 10:**
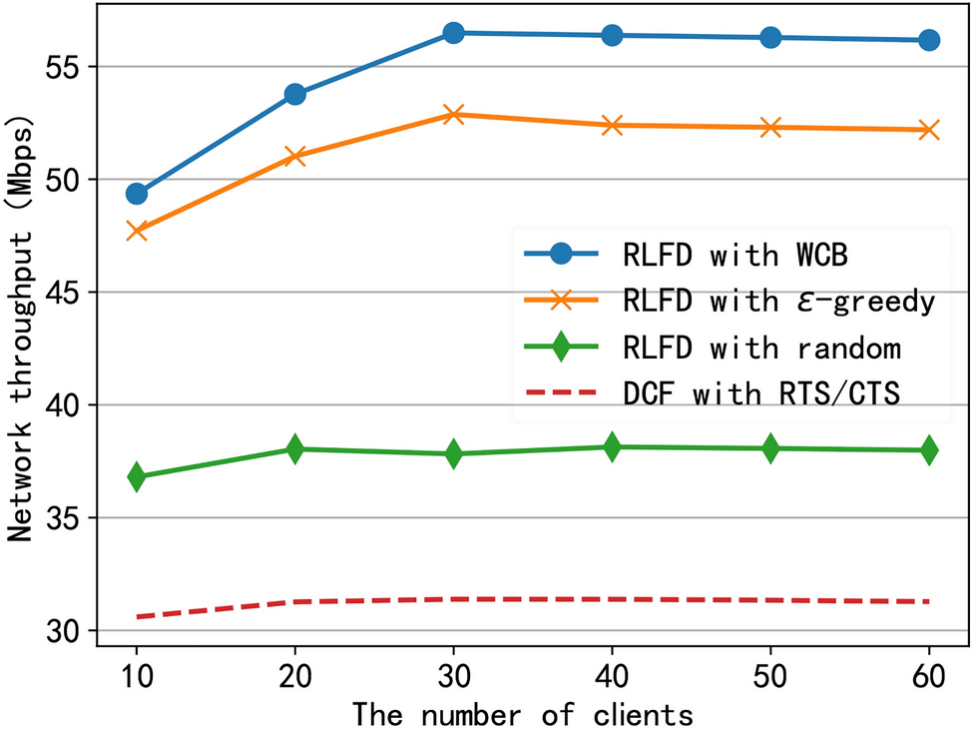
The network throughput with different numbers of clients.

#### Network throughput

To evaluate the performance of RLFD, we conducted simulation tests on the network throughput for RLFD under different network sizes. The network throughput includes both uplink and downlink throughput. For the uplink throughput, we use the calculation results based on the protocol model. For the downlink throughput, we use the average of 100 simulation results measured after algorithm convergence. As shown in Fig. 10, we compared the network throughput of RLFD using the three algorithms with the IEEE 802.11 DCF protocol. It can be seen that when the number of clients is 30, compared to IEEE 802.11 DCF with RTS/CTS protocol, RLFD with random selection strategy can increase the network throughput by 21%, RLFD with $\epsilon$-greedy algorithm can improve the network throughput by 68%, and RLFD using our proposed WCB algorithm can improve the network throughput by 80%. In addition, as the number of clients increases, the network throughput of RLFD first increases and then decreases, while that of the IEEE 802.11 DCF protocol in half-duplex mode gradually decreases. The reason is that with the increase of clients in the network, there are more full-duplex opportunities, resulting in improved network throughput, which is also proved by the results shown in Fig. 7. On the other hand, the increase in network nodes exacerbates channel competition between nodes, resulting in a decrease in network throughput.

## Discussion

The effectiveness of the proposed RLFD protocol was preliminarily verified through simulation experiments. However, in order to apply this protocol to real networks, there are still some issues that need to be considered:

- *Scalability* Although the simulation results have verified that the RLFD protocol can maintain stable network performance as the number of clients in the network increases, the state space that the RLFD protocol needs to handle will also increase accordingly. Especially, in real-world networks, clients may frequently join and leave, which can affect the performance of the RLFD protocol. The protocol’s reliance on learning optimal policies may be disrupted by frequent topology changes, requiring continuous relearning and adaptation.
- *Computational complexity* The RLFD protocol uses the WCB algorithm, which involves some computations for updating probabilities and making decisions based on the Beta distribution. Although the algorithm is a probability-based algorithm, and compared to some complex optimization algorithms, its computational complexity is relatively low, some resource-constrained devices may still not be suitable for deployment.
- *Real-time processing* In the protocol, when a node preempts the channel, AP should determine the asymmetric transmission in real-time. Considering that the packet transmission and transmission scheduling in the RLFD protocol are decoupled, the optimal scheduling policy can be calculated in advance and put it in a table. When there are new transmissions that need to be scheduled, we can directly obtain optimized scheduling results by looking up the table, particularly in environments with high packet rates or large numbers of nodes.
- *Hardware limitations* To deploy the RLFD protocol, the AP needs to support full-duplex radio. And the protocol assumes perfect self-interference cancellation (SIC) techniques can be applied in the full-duplex transmission. However, in practice, SIC is challenging to implement perfectly, and residual interference can degrade performance. The uplink reception in an asymmetric transmission may be impacted and we need adjust the data rate for uplink transmission accordingly.

## Conclusions

This paper presents a reinforcement-learning full-duplex MAC protocol called RLFD. We first investigate the full-duplex transmission scheduling problem under dynamic communication environments, and the problem is theoretically formulated as a non-stationary multi-armed bandit problem. Thus, we develop a window-constraint Bayesian algorithm to solve it, in which we introduce the LAF to improve the convergence and use window constraints to improve the adaptability in the dynamic communication environment. To effectively carry out the optimal scheduling policy and avoid hidden/exposed terminal problems, we also design a two-way handshake transmission mechanism and provide an analytical model to analyze the network performance. The numerical and simulation results show that RLFD can effectively improve the network throughput as compared to IEEE 802.11 DCF protocol. And with the WCB algorithm, it can maintain a good performance in a dynamic communication environment. In future work, we will further optimize the proposed protocol through adaptive transmit power control. By adjusting the transmit power, full-duplex transmission opportunities can be optimized, thereby improving the performance of full-duplex wireless networks.

## Data Availability

The datasets used and analyzed during the current study available from the corresponding author on reasonable request.

## List of symbols

*N*: The number of clients in the wireless network
$R^i_j$: The reward for the asymmetric transmission $S_i\rightarrow AP \rightarrow S_j$
$L^i_j$: The regret for the asymmetric transmission $S_i\rightarrow AP \rightarrow S_j$
$c_{ij}$: The SINR value at client $S_j$ in the asymmetric transmission $S_i\rightarrow AP \rightarrow S_j$
$c_{max}$: The maximum SINR value in the wireless communication
$\varpi$: The window-constraint value
$\rho$: The scaling factor when $\alpha + \beta > \varpi$
$P_{tx}$: The transmission probability for a client in a time slot
$P^a_{tx}$: The transmission probability for the AP in a time slot
*W*: The minimum contention window value for a client
$2^mW$: The maximum contention window value for a client
$W_a$: The minimum contention window value for the AP
$2^{m_a}W_a$: The maximum contention window value for the AP
*p*: The conditional conflict probability for a client
$p_a$: The conditional conflict probability for the AP
$P_{tr}$: The probability of at least one node (cliet or AP) initiating a transmission in the network
$P_{ap}$: The probability of only the AP attempting to initiate a transmission
$P_{client}$: The probability that only one client obtains the channel
$P_c$: The conflict probability of two or more nodes attempting to initiate transmission simultaneously
$T^{rts}_c$: Transmission time loss when two or more clients confilict at the handshake stage
$T^{frts}_c$: Transmission time loss when AP and clients confilict at the handshake stage
$\eta$: The payload ratio, means the improvement by utilizing full-duplex transmission
